# An introductory course to improve surgical core competencies of young hepatobiliary surgeon

**DOI:** 10.1186/s12909-026-08590-4

**Published:** 2026-01-14

**Authors:** Lei Wang, Xiao Hu, Ming Cao, Bin Zhang, Hui Hou

**Affiliations:** Department of General Surgery, The Second Affiliated Hospital of Anhui Medical University, No. 678, Furong Road, Hefei City, 230601 Anhui Province China

**Keywords:** Surgical education, CT interpretation, Hepatic anatomy, Surgical planning

## Abstract

**Introduction:**

Traditional teaching methods often fall short in addressing the challenges posed by new technologies that reveal complex anatomical details. The widespread adoption of laparoscopy and three-dimensional (3D) reconstruction in liver surgery has significantly impacted clinical education. A key focus—and ongoing difficulty—in this setting is training junior surgeons in computed tomography (CT) interpretation and surgical planning. To address this gap, we developed and implemented a structured introductory training program.

**Methods:**

In this study, our course was designed with three distinctive features: (1) integration of teaching content into routine diagnostic and therapeutic workflows; (2) the purpose of teaching was achieved by measuring the relationship between lesions and fixed anatomical landmarks using tools on CT images and during surgery; and (3) incorporation of a dedicated medical imaging and surgical video database to support daily self-practice and assessment.

**Results:**

Between January and December 2024, 107 trainees completed the course and were invited to participate in a post-training survey, and 91 responses were collected. Feedback indicated strong satisfaction with the proposed teaching model. Respondents highlighted the value of the image and video database as an effective learning resource. In addition, performing anatomical landmark labeling and conducting multi-dimensional measurements were reported to reinforce the understanding of hepatic anatomy and CT interpretation.

**Conclusions:**

This study outlines the design and initial implementation of an introductory course aimed at improving core surgical competencies among early-career hepatobiliary surgeons. Survey results support its educational utility and acceptability, providing a basis for further curricular refinement and broader adoption.

**Supplementary Information:**

The online version contains supplementary material available at 10.1186/s12909-026-08590-4.

## Introduction

 In China, a nationwide standardized residency training system (Stage 1) was established in 2014. Subsequently, in accordance with the training standards and requirements for different specialties, individuals seeking subspecialization in liver surgery (a subspecialty of general surgery) are required to complete a 2-year specialized training program in liver surgery as hospital-based faculty members (Stage 2) [1, 2]. These programs primarily emphasize general surgical techniques and procedures [2, 3]. The core competencies and teaching objectives include mastering hepatic anatomy and developing preliminary surgical planning abilities [4, 5].

Due to the complex anatomical structure of the liver and the technical challenges of hepatic operations, thorough preoperative evaluation and surgical planning are essential. Achieving these competencies requires extensive clinical exposure to complete diagnostic and therapeutic cases [6]. However, liver surgery education remains difficult. The liver’s irregular wedge-shaped morphology, variable anatomical landmarks, and intricate vascular and biliary systems require strong spatial reasoning, which many beginners lack.

Because of individual anatomical variations, preoperative imaging—especially contrast-enhanced computed tomography (CT)—is indispensable. Sequential observation of CT slices helps form a mental three-dimensional (3D) reconstruction [7]; however, this mental visualization is often indistinct and prone to error, particularly for novices. Therefore, auxiliary tools that render spatial relationships more concrete, accurate, and assessable are required. CT interpretation in liver surgery must not only localize lesions but also guide surgical planning—determining safe resection margins, identifying vascular variations, selecting optimal transection planes, and assessing relationships between lesions and major vessels [8, 9]. Nevertheless, few studies have proposed methods to improve CT interpretation skills in liver surgery.

Traditional teaching methods that rely on theoretical lectures and static images are insufficient for conveying the complexity of liver anatomy and the dynamic nature of surgical planning. To address this, our department has implemented an innovative introductory training program that integrates education into routine clinical workflows. The program leverages an integrated image and video database, emphasizes multi-dimensional measurement to deliver a more intuitive and practical learning experience, and aims to enhance trainees’ understanding of anatomy, proficiency in CT interpretation, and surgical spatial reasoning skills.

This study describes the implementation framework of this curriculum, discusses practical insights from multi-dimensional measurement-based teaching using image and video anatomical data, and reports initial trainee evaluations.

## Methods

### Educational materials

#### Liver imaging and surgical video database

The database includes data from more than 100 patients, each case containing enhanced CT images, 3D reconstruction images, and laparoscopic hepatectomy videos. Data collection followed a standardized process of sorting and annotation. CT images were carefully segmented and labeled to highlight key anatomical structures and lesions. 3D reconstructions images were generated using advanced software algorithms for high-quality visualization, and surgical videos were annotated with important surgical steps and anatomical landmarks for better understanding by trainees. The database was developed by five senior surgical instructors from the department and one medical trainee, with approval from the institutional ethics committee. All patients provided informed consent for the educational use of anonymized surgical videos.

#### Smart conference Panel/Touchscreen display

This device can connect to the database and the hospital’s Picture Archiving and Communication System (PACS), enabling real-time teaching, case discussions, and remote communication, including intraoperative video transmission (Supplementary Fig. 1).

### Participants

Participants included Stage 1 standardized training residents (who had just completed undergraduate education and were in the initial phase of clinical training) and Stage 2 hepatobiliary surgery specialty trainees (who had formally the first stage of residency training and opted for hepatobiliary surgery as their subspecialty). Before the study, the two groups differed in their proficiency in general surgery fundamentals. However, both still had limited experience in liver-specific anatomy and surgical planning.

### Integrating teaching into the daily workflow

#### Stage 1: theoretical instruction

Five senior surgeons in the department give regular theoretical lectures to new trainees, and previous trainees can participate in the course more than once. Topics included the method of CT interpretation, intrahepatic and extrahepatic anatomical structures and their appearance on CT images, how to use the database, and the method of multi-dimensional measurement on a CT image using PACS tools. Additionally, hand-drawing methods for surgical planning, as well as the methods, skills, and key points that should be considered during hand-drawing have also been introduced [10–12]. Trainees with slower progress receive one-on-one tutoring focusing on simplifying complex vascular relationships, while advanced trainees explore topics such as anatomical variations and recent advances in liver surgery.

#### Stage 2: Self-Study and exercises (Optional)

The database is accessible to all medical trainees and tutors. Trainees can access the database via departmental computers through the hospital’s local area network. They are also encouraged to identify fixed extrahepatic or intrahepatic landmarks as reference points or planes, which can be used to measure the distances or angles between the lesion and landmarks, thereby improving spatial accuracy.

(1) Construct a mental 3D image by reviewing continuous CT slices (like watching an animated movie).

(2) Measure distances and angles using PACS tools, and estimate lesion depth based on slice counts.

(3) 3D reconstruction images and surgical videos can serve as valuable references. However, it is recommended to use them after learning to interpret CT images. Faster learners conduct in-depth analyses of complex cases, while those who encounter difficulties receive peer assistance and tips for efficient spatial visualization.

Trainees with a faster learning pace are encouraged to conduct in-depth analyses of special cases and explore the advantages and disadvantages of different surgical plans using database resources. For those with learning difficulties, senior trainees will be arranged for peer-to-peer assistance and sharing learning experiences and techniques, such as how to more efficiently construct three - dimensional images.

#### Stage 3: case discussion

During routine preoperative discussions [5, 13], trainees not only present the patients’ clinical data but also provide CT images of multiple consecutive layers. As a key part of preoperative evaluation, they perform multi-dimensional measurements on CT images to assess the spatial relationships between the lesion and surrounding anatomical landmarks. It is emphasized that the purpose of these measurements is to gain a better understanding of such spatial relationships. Trainees can communicate with one another during this process and try to come up with a preliminary surgical plan. To improve clarity, it is recommended to include one or more hand-drawings to illustrate the preliminary surgical procedure (optional).

The specific modular content preparation and CT interpretation competency assessment criteria are as follows:

- Mark the location and shape of the lesions. ----- Identify the lesions. (Basic).
- Accurately label the pertinent CT measurements and present them in precise proportions, such as distance measurements in four orientations (cephalic, caudal, ventral, and dorsal), along with distance or angle measurements referencing appropriate extrahepatic or intrahepatic landmarks ---- Can provide more details and be able to use some important landmarks. (Good)
- Identify and annotate the major adjacent or nutrient vessels and delineate the vessels surrounding the lesion that require preservation or removal, along with their respective distances from the lesions. ---- Can propose a preliminary surgical plan. (Excellent)

Finally, tutors offer their insights and recommendations for improvement. The components of the evaluation include whether anatomical structures are correctly identified, whether landmarks are reasonably chosen, whether measurement data are accurate, and whether the surgical plan is precise.

When trainees show difficulties in understanding during the case discussion, the tutors guide them to start with basic anatomical knowledge, gradually analyze the relationship between the lesion and anatomical landmarks, and provide more similar cases for comparative learning. For outstanding trainees, the tutors encourage them to put forward innovative surgical planning ideas and lead in-depth discussions during the process.

#### Stage 4: surgical practice

In this department, laparoscopy has been maturely applied in most partial liver resection operations, including laparoscopic right hemihepatectomy, right anterior lobectomy, laparoscopic caudate lobectomy, and other relatively difficult operations. Trainees participate in the operation as assistants in corresponding cases. Real-time intraoperative imaging enhances understanding of anatomy, while intraoperative measurements (using ultrasound or other instruments) validate preoperative spatial assessments. Comparing pre- and intraoperative data reinforce learning and increased engagement.

During surgery, according to the differences in trainees’ basic knowledge, beginners are given more opportunities to observe basic operations and identify anatomical structures. Trainees with some experiences are allowed to participate in more complex operation and receive targeted guidance. For example, when dealing with blood vessels, trainees are taught how to perform precise operations based on the preoperative measurement data.

#### Stage 5: learner evaluation and statistical methods

Trainees who had completed more than ten full case cycles were invited to fill out an anonymous survey focusing on their learning experiences and perceptions of the teaching process. The questionnaire was designed based on a comprehensive review of relevant medical education literature and input from experienced liver surgeons. Prior to administration, the survey was reviewed by a team member with survey design experience, and a pilot test was conducted among a small group of trainees to identify and rectify potential issues with question clarity or ambiguity, both of which aimed to ensure a comprehensive and accurate collection of trainees’ feedback on the teaching process. The questionnaire covered aspects such as overall satisfaction with the teaching model, satisfaction with each teaching link (e.g., theoretical teaching, case discussion), user experience with different teaching tools (e.g., the database, hand-drawing), and perceptions of self-perceived ability improvement. The study was approved by the Ethics Committee of the Second Affiliated Hospital of Anhui Medical University and conducted according to the Declaration of Helsinki. The teaching administration prohibits faculty and students from disseminating any operation videos or images for non-teaching purposes. Responses were measured using a Likert scale [14] and summarized as median (interquartile range, IQR). Descriptive statistics were applied. Future research will consider incorporating objective skill assessments, such as operational evaluations based on virtual surgery platforms and surgical simulation planning assessments, to evaluate teaching effectiveness more comprehensively from multiple dimensions.

## Results

**Fig. 1:**
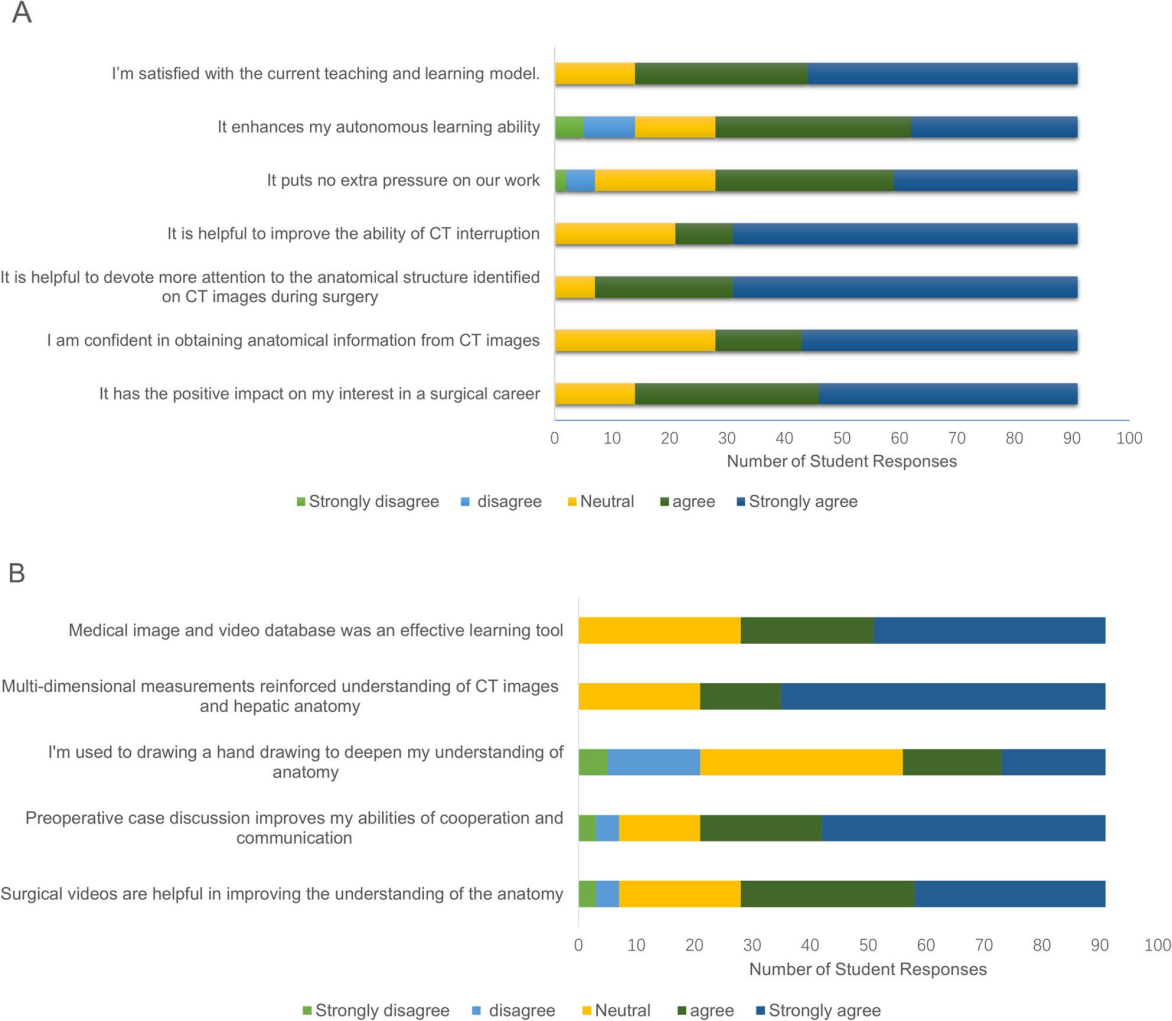
Student’s perspective on the current teaching-learning model and teaching details(*n* = 91). Based on a 5-point Likert scale, where 1 is strongly disagree and 5 is strongly agree

From January 2024 to December 2024, a total of 107 trainees completed the learning process and received the questionnaire. Of these, 96 (89.7%) were Stage 1 standardized training trainees, among whom 16 were professional postgraduates majoring in hepatic surgery—these trainees had a clear professional orientation, giving them greater advantages in learning initiative and interest. The remaining 11 were Stage 2 hepatobiliary surgery specialty trainees (Supplementary Fig. 2). Ultimately, 91 (83 males) responded to the questionnaire, as summarized in Fig. 1; Tables 1 and 2. All trainees were deemed by their teaching supervisors to meet all the requirements of the teaching milestones in the final assessment upon completion of their clinical rotation.

**Table 1:** Student's perspective on the current teaching and learning model (n=91)

	Strongly disagree + disagree *n* (%)	Neutral *n* (%)	Strongly agree +agree *n* (%)	Median (IQR)^a^
I’m satisfied with the current teaching and learning model.	0 (0)	14 (15)	77 (85)	5 (4，5)
It enhances my autonomous learning ability	14 (15)	14 (15)	63 (69)	4 (3，5)
It puts no extra pressure on our work	7 (8)	21 (23)	63 (69)	4 (3，5)
It is helpful to improve the ability of CT interruption	0 (0)	21 (23)	70 (77)	5 (3.5，5)
It is helpful to devote more attention to the anatomical structure identified on CT images during surgery	0 (0)	7 (8)	84 (92)	5 (4.5，5)
I am confident in obtaining anatomical information from CT images	0 (0)	28 (31)	63 (69)	4 (3，5)
It has the positive impact on my interest in a surgical career	0 (0)	14 (15)	77 (85)	4 (4，5)

Based on a 5-point Likert scale, where 1 is strongly disagree and 5 is strongly agree

**Table 2:** Student's perspective on teaching details (*n*=91)

	Strongly disagree + disagree *n* (%)	Neutral *n* (%)	Strongly agree +agree *n* (%)	Median (IQR)
Medical image and video database was an effective learning tool	0 (0)	28 (31)	63 (69)	4 (3，5)
Multi-dimensional measurements reinforced understanding of CT images and hepatic anatomy	0 (0)	21 (23)	70 (77)	5 (3.5，5)
I'm used to drawing a hand drawing to deepen my understanding of anatomy	21 (23)	35 (38)	35 (38)	3 (2.5，4)
Preoperative case discussion improves my abilities of cooperation and communication	7 (8)	14 (15)	70 (77)	4 (3.5，5)
Surgical videos are helpful in improving the understanding of the anatomy	7 (8)	21 (23)	63 (69)	4 (3，5)

Based on a 5-point Likert scale, where 1 is strongly disagree and 5 is strongly agree

The vast majority of respondents (85%, with a median of 5 and an interquartile range [IQR] of 4–5) expressed satisfaction with the current teaching and learning model. Most respondents (69%, median = 4, IQR = 3, 5) concurred that this model bolsters their autonomous learning capabilities without imposing additional work-related stress. A substantial majority (77%, median = 5, IQR = 3.5, 5) reported improved CT interpretation skills. For example, in a case involving a patient with a complex liver tumor near the hepatic hilum, trainees who participated in this teaching program were able to accurately discern the tumor’s relationship with adjacent blood vessels and bile ducts on CT images. They could also precisely measure distances and angles—skills that have a direct bearing on surgical planning. This indicates that the teaching program effectively sharpens the trainees’ CT interpretation skills in real-world clinical settings. Additionally, 92% of the respondents reported that the program improved their awareness of the anatomical structures identified on CT images during surgery.

In terms of teaching scenes and teaching details, a majority of respondents agreed or strongly agreed with the statement that medical image and video databases were an effective learning tool (69%, median = 4, IQR = 3, 5), labeling landmarks and making multi-dimensional measurements reinforced understanding of CT images and hepatic anatomy (77%, median = 5, IQR = 3.5, 5), preoperative case discussion improved their abilities of cooperation and communication (77%, median = 4, IQR = 3.5, 5), and surgical videos were helpful in improving the understanding of the anatomy (69%, median = 4, IQR = 3, 5). However, only 38% of respondents (median = 3, IQR = 2.5, 4) agreed or strongly agreed that they used to draw a hand-drawing to deepen their understanding of anatomy.

**Fig. 2:**
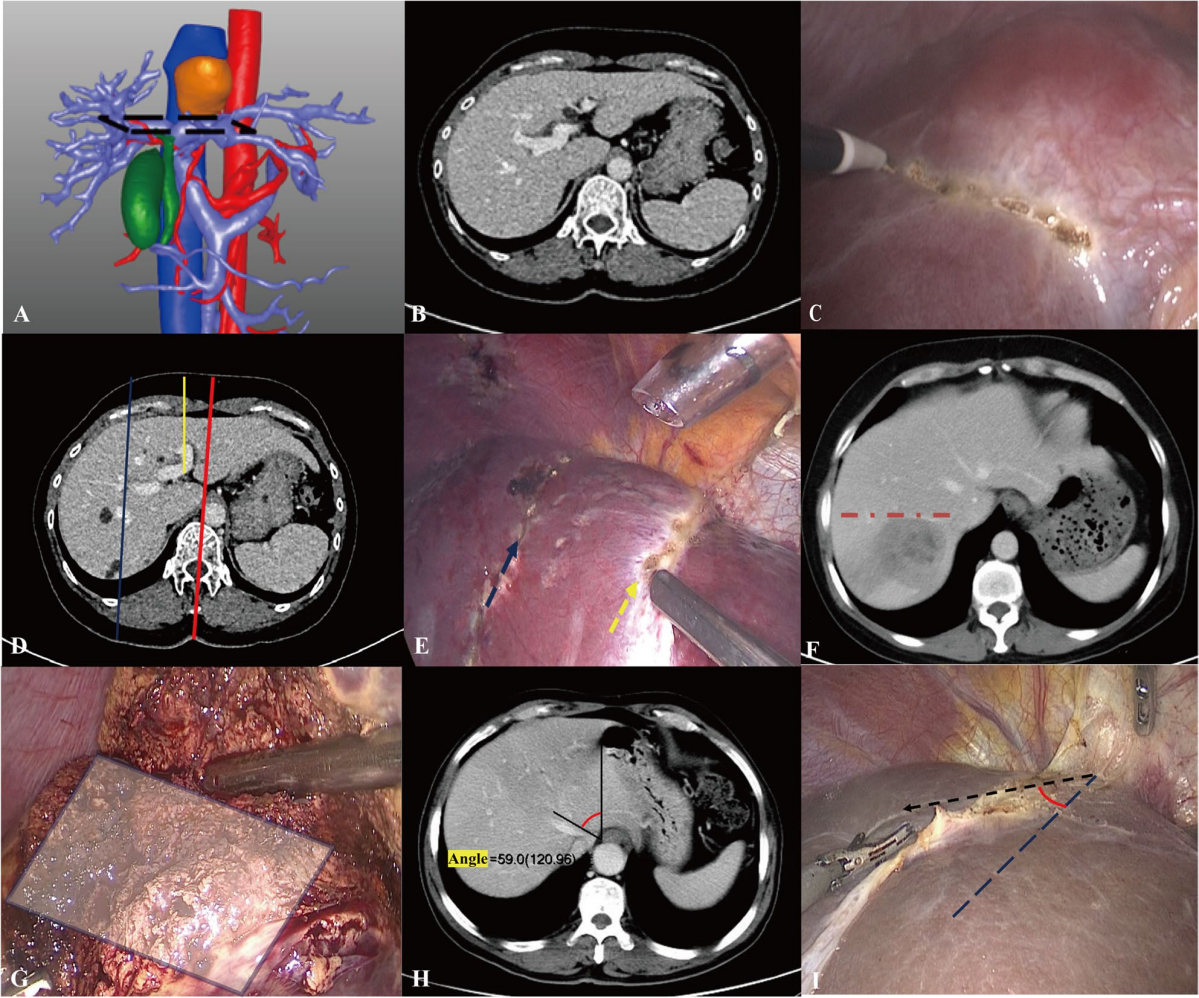
Some extrahepatic and intrahepatic anatomical landmarks considered to be characteristic in this course

Trainees have also learned to identify critical intrahepatic and extrahepatic anatomical landmarks, such as the hepatic veins, ligamentum venosum, gallbladder, and Rouvière’s sulcus [15–18], and to adopt individualized surface projection markers to enhance the precision of surgical localization. As illustrated in Fig. 2A–C, the first porta hepatis and its projection onto the diaphragmatic surface of the liver serve as important reference plane, enabling trainees to orient themselves with respect to the internal hepatic structures and to determine the optimal surgical approach. In (A), the target plane is highlighted by a black dashed rectangle, with (B) and (C) showing its cross-sectional level on CT imaging and its projected location on the liver surface during surgery, respectively. In Fig. 2D–E, the linea mediana ventralis (red line), the sagittal portion of the portal vein ligamentum teres hepatic (yellow line), midclavicular line (blue line) and their surface projections (dotted arrows), helps trainees achieve more accurate localization of intrahepatic lesions. Through this process of identifying and measuring anatomical landmarks, trainees effectively strengthened their spatial understanding of the hepatic anatomy and improved their surgical planning capabilities. Moreover, the angle between the plane formed by the right hepatic vein and the patient’s horizontal plane (Fig. 2F) provides key information for laparoscopic right posterior hepatectomy. When the right hepatic vein runs approximately parallel to the horizontal plane (red dotted line), transection of the hepatic parenchyma from the caudal to the cephalad direction along this plane (shaded gray area) facilitates an easier and safer operation (Fig. 2G). Similarly, the angle between the middle hepatic vein and the right margin of the esophagus (Fig. 2H–I) offers additional orientation cues for intrahepatic dissection.

**Fig. 3:**
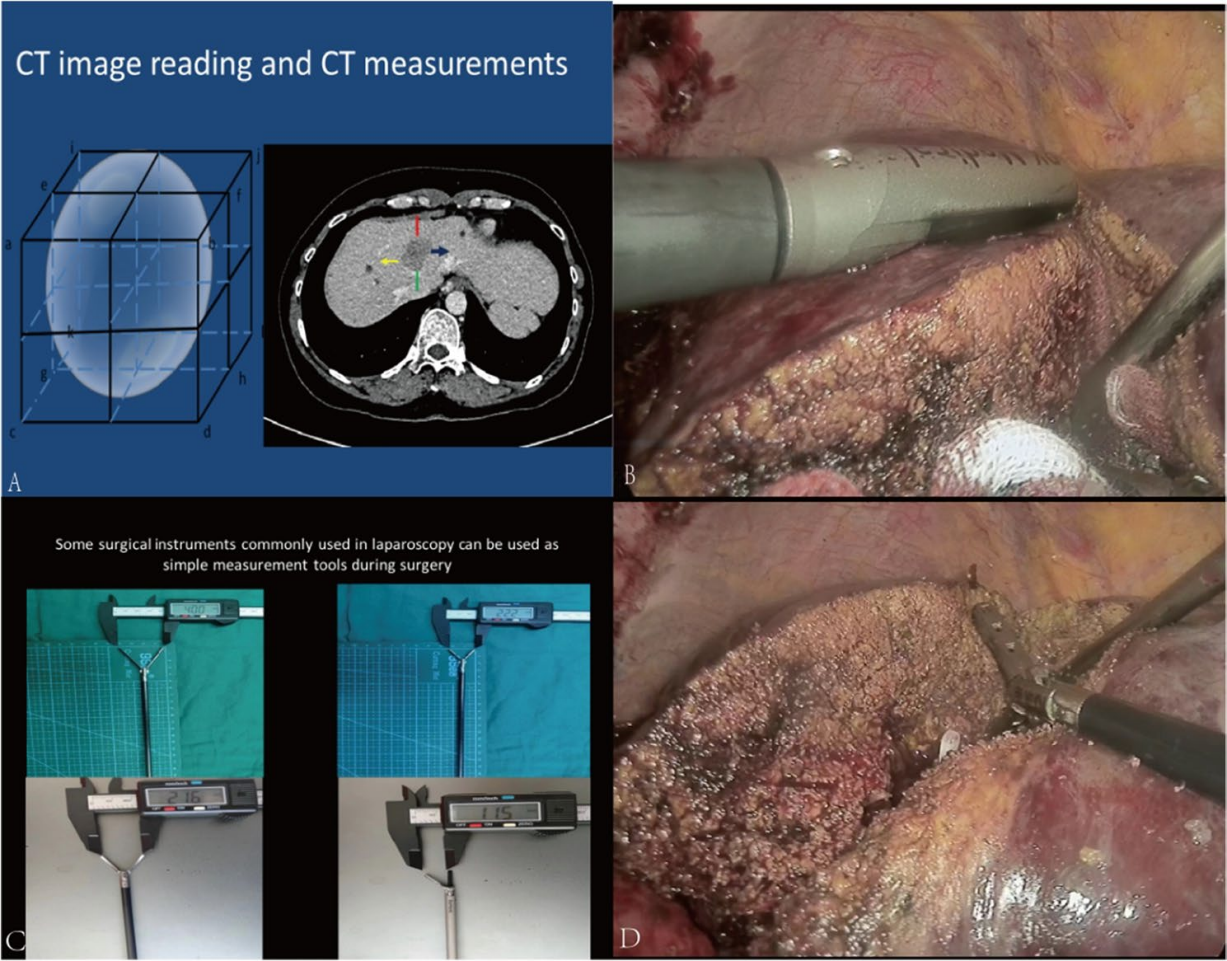
Intraoperative measurements were made by ultrasound or some instruments. 3 **A** Schematic diagram of multi-dimensional measurement on CT images; 3**B**. Intraoperative ultrasound was used for intraoperative measurements; 3 **C**-**D**. Some standard surgical instruments can be used as simple and quick measurements during surgery

Intraoperatively, several standardized surgical instruments were also employed as convenient tools for real-time measurements (Fig. 3). As shown in Fig. 3A, the schematic diagram of multi - dimensional measurement on CT images shows how to measure the distance between the lesion and anatomical landmarks from different orientations, providing quantitative support for preoperative planning. As shown in Fig. 3B, intraoperative ultrasonography was utilized to assess the depth of lesions in real time, helping surgeons precisely control the dissection depth and operative plane. Standard surgical instruments, such as laparoscopic grasping forceps, as shown in Fig. 3C–D, can also serve as quick and practical measuring tools during surgery. By using the length of these instruments as reference scales, surgeons can conveniently estimate distances between intrahepatic structures, ensuring the accuracy and safety of operative maneuvers. This combination of preoperative imaging-based measurement and intraoperative quantitative validation not only enhances surgical precision but also reinforces trainees’ understanding of anatomical-spatial relationships during hepatobiliary surgery.

## Discussion

During departmental rotations, trainees are trained to be responsible for the entire process of patient management under the supervision of senior attending surgeons, who serve as chief surgeons and primary decision-makers for diagnosis and treatment [2, 3]. In addition, junior surgeons are required to complete detailed medical documentation and participate regularly in a variety of institutional training and assessment activities. They face dual pressures—clinical workload and academic research obligations—while simultaneously preparing for future qualification examinations and employment opportunities [2, 3]. Additionally, in current medical education and training, the hierarchy of trainees presents a “pyramid” distribution: Stage 1 serves as a critical starting point for physicians to engage in clinical practice, and the number of trainees at this stage is far greater than that in Stage 2. In this study, the target population exhibited significant differences in their specialized foundational knowledge, clinical skills, and learning motivation, additionally, this group requires high levels of self-directed learning and strong initiative. This “pyramid” trainee structure determines that the course must be inclusive and universally applicable to cover the learning needs of trainees at different levels. At the same time, it must retain a certain degree of flexibility to reflect differentiation in teaching details. These combined demands pose significant challenges to the design of an effective and sustainable training curriculum. Encouraging early engagement empowers young trainees, facilitates peer interaction and mentorship, and supports career development through networking and collaborative learning opportunities [17].

Current evidence indicates that the early years of medical training are a critical determinant of long-term career success [19, 20]. An introductory curriculum that promotes continuous professional development should incorporate several essential elements: surgical coaching [21]; skill development in translating theoretical knowledge into practical application [22, 23]; fostering cooperation and communication skills [22, 24, 25]; integrating multimodal learning (visual, auditory, and kinesthetic modalities); and establishing an efficient, learner-centered platform to minimize burnout [20, 23, 26]. However, relatively few studies have investigated long-term methods for assessing knowledge acquisition or surgical skill development. The existing literature is largely constrained by methodological limitations, with most studies relying on self-reported questionnaires or interviews to evaluate the effects of professional training [23]. Objective, longitudinal data remain scarce.

A substantial majority of respondents (85%, median = 5, IQR = 4, 5) expressed overall satisfaction with the current teaching and learning model. Most respondents (69%, median = 4, IQR = 3, 5) agreed that the training enhanced their autonomous learning ability without adding to their workload. Additionally, 69% (median = 4, IQR = 3, 5) considered the integrated medical image and video database to be an effective learning tool, and an equal proportion agreed that surgical videos significantly improved their anatomical understanding. In China, the minimum number of operative cases and the opportunities to serve as chief surgeons or first assistants during residency are lower than those in Western training systems [2, 6]. This gap is particularly evident in high-risk, highly-specialized fields, such as hepatobiliary surgery. Limited surgical exposure makes it challenging for trainees to meet training requirements. To address this, numerous virtual anatomy resources—including three-dimensional reconstruction images and video/images from surgical procedures, have been incorporated into modern medical education [25–28]. Unlike static two-dimensional textbook images, dynamic 3D tools allow visualization of anatomical structures from multiple perspectives. In hepatic surgery, 3D reconstruction based on contrast-enhanced CT is widely used for the preoperative assessment of complex cases, assisting in resection planning, and residual liver volume estimation. Moreover, the increasing adoption of laparoscopic hepatectomy driven by advances in surgical instruments and imaging technology has greatly expanded the educational potential of real-time video capture. High-definition intraoperative recordings and remote streaming, paired with an expanded field of view that enhances visualization of crucial anatomical structures, provide both synchronous and asynchronous learning opportunities. When systematically organized, these materials constitute a powerful educational resource for understanding surgical anatomy. In our program, CT images, 3D reconstruction images, and visualized surgical videos were integrated into a comprehensive database designed for repeated, self-paced studies. Our long-term goal is to develop a cloud-based platform that allows trainees to access these materials remotely via personal devices, thereby enhancing the flexibility and accessibility of surgical learning.

A majority of respondents (77%, median = 5, IQR = 3.5, 5) endorsed that labeling anatomical landmarks and performing multi-dimensional measurements strengthened their understanding of CT imaging and hepatic anatomy. Similarly, 77% (median = 4, IQR = 3.5–5) found that preoperative case discussions improved teamwork, cooperation, and communication skills. In clinical practice, locating intrahepatic lesions relative to fixed anatomical or surface landmarks is a common and essential surgical skill [10, 15–18, 29]. Although 3D visualization technologies are now widely used in anatomy education [26, 27], proficiency in CT interpretation—particularly dynamic contrast-enhanced CT—remains a fundamental competency for hepatobiliary surgeons. Early training of this skill is crucial for the future career development of junior surgeons.

Quantitative measurements of spatial parameters such as depth, distance, and angle provide valuable support for developing spatial reasoning and surgical planning abilities. In our evaluation system, greater emphasis was placed on textual interpretation and quantitative analysis of CT images. Hand-drawing was primarily encouraged as a supplementary self-study and communication tool. However, only 38% of respondents (median = 3, IQR = 2.5, 4) endorsed using hand-drawing to deepen their anatomical understanding. This low adoption may be attributed to limited drawing skills among trainees and the relatively short training duration. For beginners lacking artistic training, producing accurate 3D sketches within a short timeframe can be challenging and may even increase their workload rather than facilitating communication.

This study had several limitations. Course caters to trainees at multiple levels, and those with strong learning motivation and interest may benefit more significantly. However, the pyramid distribution of trainee levels and sample size requirements (particularly the small number of Stage 2 trainees) necessitated the inclusion of multiple groups in the survey, which may have introduced selection bias and prevented a comprehensive reflection of the diverse backgrounds and learning abilities of different trainees. Additionally, in accordance with the teaching objectives, the assessment criteria for Stage 1 and Stage 2 trainees were not uniform, making direct comparisons between the two groups difficult. The current study only explored trainees’ acceptance of the course and their preliminary feedback, which also constitutes a limitation of this research.

Additionally, several aspects of the curriculum require further refinement. Hand-drawing training was not explicitly emphasized, and we did not track individual usage time within the database—both factors that could have influenced learning outcomes. Furthermore, the short teaching duration might have limited the trainees’ mastery of complex anatomical and surgical concepts. Given that different surgical types (e.g., complex hilar vs. segmental resections) vary in difficulty and learning emphasis, the current teaching model may need to be adapted accordingly. Future studies should consider these variables to further optimize the program and enhance its effectiveness.

## Conclusion

In conclusion, a comprehensive database integrating two-dimensional CT images, three-dimensional reconstructions, and surgical videos from previous clinical cases provides an effective educational tool for teaching anatomy and fostering surgical reasoning in hepatobiliary training. Considering the widespread use of laparoscopic and 3D technologies in modern liver surgery, incorporating measurement-based spatial localization into the early teaching curriculum represents a practical and efficient strategy for developing the foundational competencies of junior hepatobiliary surgeons.

## Supplementary Information

Supplementary Material 1: Supplementary Fig.S1: A platform for medical teaching and case discussion

Supplementary Material 2: Supplementary Fig.S2: Composition of trainees in hepatobiliary surgery training course

## Data Availability

The datasets used and/or analysed during the current study are available from the corresponding author upon reasonable request.

## Abbreviations

3D reconstruction: three-dimensional reconstruction
CT: computerized tomography
PACS: picture archiving and communication system
