# Supplementary figures and images for: An introductory course to improve surgical core competencies of young hepatobiliary surgeon
Source: BMC Med Educ. 2026 Jan 14;26:231. doi: 10.1186/s12909-026-08590-4 (PMC12888341; doi:10.1186/s12909-026-08590-4)

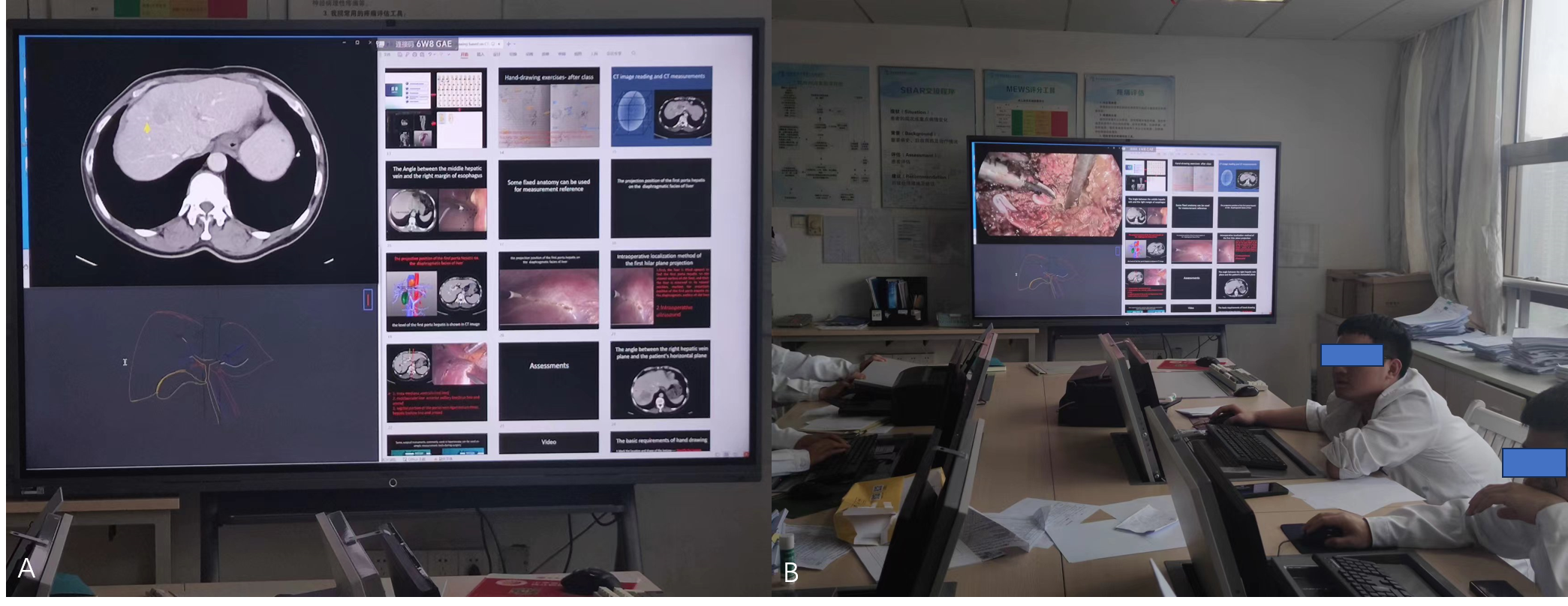

Supplement: Supplementary file 1 — Supplementary Material 1: Supplementary Fig.S1: A platform for medical teaching and case discussion [file 12909_2026_8590_MOESM1_ESM.tif]

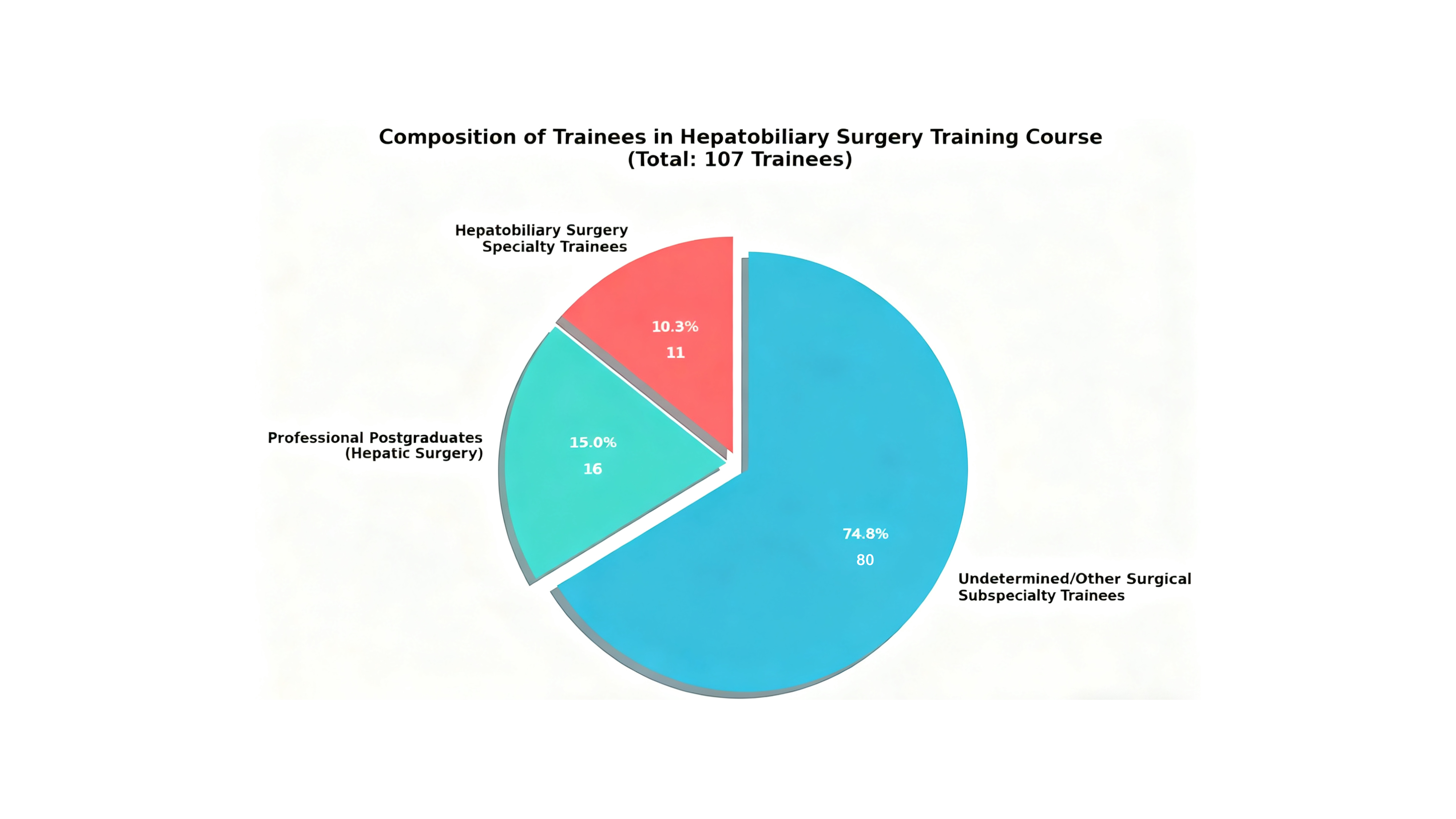

Supplement: Supplementary file 2 — Supplementary Material 2: Supplementary Fig.S2: Composition of trainees in hepatobiliary surgery training course [file 12909_2026_8590_MOESM2_ESM.tif]
